# Loquat (*Eriobotrya japonica*) Is a New Natural Host of Tomato Mosaic Virus and Citrus Exocortis Viroid

**DOI:** 10.3390/plants13141965

**Published:** 2024-07-18

**Authors:** Chengyong He, Lingli Wang, Yarui Li, Kangyu Zhou, Ke Zhao, Dong Chen, Jing Li, Haiyan Song, Meiyan Tu

**Affiliations:** 1 Institute of Horticulture, Sichuan Academy of Agricultural Sciences, Chengdu 610066, China; hchyong2019@163.com (C.H.); abl13272755@126.com (L.W.); lyrui1920@163.com (Y.L.); zhou13952253516@163.com (K.Z.); zhaoke0607@163.com (K.Z.); cd13219071209@163.com (D.C.); lijing412@126.com (J.L.); shy19913@163.com (H.S.); 2 Key Laboratory of Horticultural Crop Biology and Germplasm Creation in Southwest China, Ministry of Agriculture and Rural Affairs, Chengdu 610066, China; 3 College of Horticulture, Anhui Agricultural University, Hefei 230036, China

**Keywords:** loquat, ToMV, CEVd, new natural host, first report

## Abstract

Loquat leaves exhibiting obvious yellowing, blistering, mosaic, leaf upward cupping, crinkle, and leaf narrowing were identified in Panzhihua City, Sichuan Province, China. High-throughput sequencing (HTS) with the ribo-depleted cDNA library was employed to identify the virome in the loquat samples; only tomato mosaic virus (ToMV) and citrus exocortis viroid (CEVd) were identified in the transcriptome data. The complete genome sequence of ToMV and CEVd were obtained from the loquat leaves. The full-length genome of the ToMV-loquat is 6376 nt and comprises four open reading frames (ORFs) encoding 183 kDa protein, RNA-dependent RNA polymerase (RdRp), movement protein (MP), and coat protein (CP), respectively. A pairwise identity analysis showed that the complete sequence of the ToMV-loquat had a nucleotide identity between 98.5 and 99.3% with other ToMV isolates. A phylogenetic analysis indicated that ToMV-loquat was more closely related to ToMV-IFA9 (GenBank No. ON156781). A CEVd sequence with 361 nt in length was amplified based on the HTS contigs, sequence alignment indicated CEVd-loquat had the highest identity with the strain of CEVd-Balad (GenBank No. PP869624), phylogenetic analysis showed that CEVd-loquat was more closely related to CEVd-lettuce (GenBank No. ON993891). This significant discovery marks the first documentation and characterization of ToMV and CEVd infecting loquat plants, shedding light on potential threats to loquat cultivation and providing insights for disease management strategies.

## 1. Introduction

Loquat (*Eriobotrya japonica*), a member of the family Rosaceae, is an evergreen fruit tree originating from China. Revered for its delectable fruits and esteemed medicinal attributes, the loquat has been cherished for over 2100 years in China, boasting a rich cultivation legacy. In China, the epicenter of loquat cultivation, the fruit holds paramount importance, reigning as the world’s foremost producer with an annual yield surpassing 1 million tons. This robust production not only sustains local economies but also serves as a vital resource in global fruit markets. Fruit trees are susceptible to virus infection; however, research on the loquat virus was not initiated until 2019, with the first report of apple stem grooving virus (ASGV) and apple chlorotic leaf spot virus (ACLSV) [1]. To date, only five viral pathogens have been reported to infect loquat, including ASGV [1,2], ACLSV [1], loquat virus A (LoVA) [3], apple stem pitting virus (ASPV) [4], and apple hammerhead viroid (AHVd) [2].

Tomato mosaic virus (ToMV) belongs to the genus *Tobamovirus* of the family *Virgaviridae*. Its genome consists of a single-stranded, positive-sense RNA molecule approximately 6.4 kb in length. This RNA encodes at least four proteins crucial for the virus’s lifecycle and pathogenicity. 126 kDa protein and 183 kDa protein are viral replicates, and the latter is predicted to be synthesized through the read-through product of the 126 kDa protein. 30 kDa protein and 17.5 kDa protein encode movement protein (MP) and coat protein (CP), respectively. ToMV is a globally widespread pathogen that infects a wide range of commercially important crops from the Solanaceae and Cruciferae families. These include tomatoes, eggplants, tobacco, and radishes. The virus is known to be transmitted via seed and mechanical friction; it has also been reported to be transmitted through irrigation water [5] and causes variables symptoms, depending on the specific cultivar of the host plant, including the mosaic, vein clearing, puckering, and distortion of leaves, flower dropping, and stunting and brown necrosis on the pedicles, stems, and sepals [6,7,8], resulting in significant economic losses due to reduced fruit quality and yield in affected crops [9,10].

Viroids are the smallest known plant pathogens, ranging in length from 246 to 401 nucleotides [11]. They are unique in nature, being composed of naked, single-stranded, non-coding circular RNA molecules. These RNA molecules exhibit a highly compact, covalently closed secondary structure and do not encode any known proteins or peptides [12,13]. Viroids are classified into the following two families: *Pospiviroidae* and *Avsunviroidae*, depending on the most recent taxonomy by the International Committee on Taxonomy of Viruses (ICTV) (https://ictv.global/taxonomy (accessed on 15 March 2024)). Citrus exocortis viroid (CEVd) belongs to the genus *Pospiviroid* of the family *Pospiviroidae.* Viroids in this family typically adopt rod-like conformations and replicate within the nucleus of their host plants [14]. CEVd is the causal agent of citrus exocortis disease, which is a bark-shelling or -scaling disorder associated with the dwarfing of trees grafted on trifoliate orange or its hybrids and induces considerable fruit yield loss in citrus crops [15,16]. Microarray analyses have shown that CEVd infection alters various physiological processes in plants, including changes in chloroplast function, cell wall structure, peroxidase activity, and symporter activities in Etrog citron (*Citrus medica* L.) [17]. Additionally, CEVd is responsible for defective ribosome biogenesis in tomatoes, thereby interfering with the translation machinery and causing ribosomal stress [18].

In this study, virus-like symptoms were observed on the leaves of three loquat plants sampled from Sichuan province, a major loquat growing area in China. High-throughput sequencing (HTS) was employed to detect the presence of viruses and viroids, and their presence was subsequently confirmed using reverse transcription polymerase chain reaction (RT-PCR). For the first time in loquats, two pathogens were identified, as follows: ToMV and CEVd. These findings highlight the presence of these pathogens in loquat plants from Sichuan, indicating potential implications for disease management and quarantine measures in loquat cultivation.

## 2. Results

### 2.1. Viruses Detected in Loquat Using High-Throughput Sequencing

**Figure 1:**
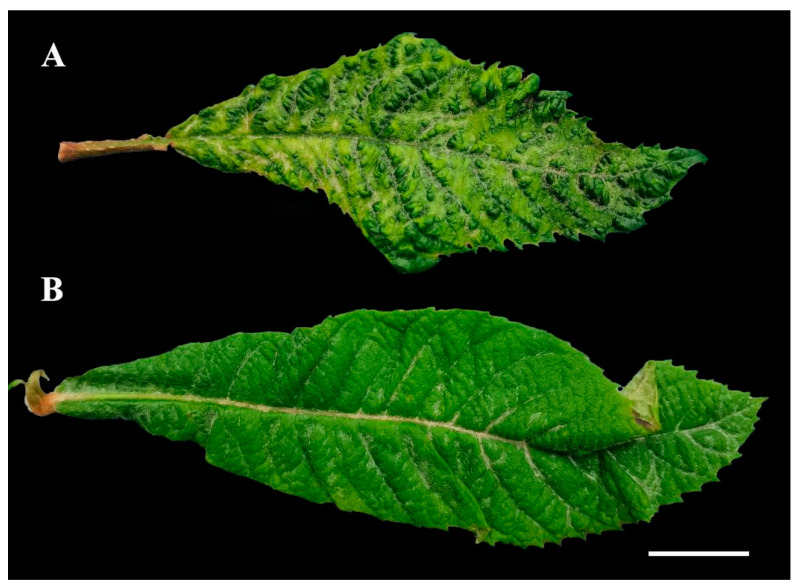
Disease symptoms of loquat. (**A**) Loquat leaf showed symptoms of yellowing, blistering, mosaic, leaf upward cupping, crinkle, and leaf narrowing. (**B**) Healthy loquat leaf. Scale bar = 2 cm.

In November 2023, loquat leaves exhibited symptoms typical of viral infection, including yellowing, blistering, mosaic, leaf upward cupping, crinkle, and leaf narrowing, were observed in Panzhihua City, Sichuan Province, China. These loquat leaves were collected and photographed (Figure 1). To identify potential viruses responsible for these symptoms, total RNAs were isolated from the loquat leaves of three different trees, and the RNAs underwent ribosomal RNA depletion, resulting in ribo-depleted RNA samples. These samples were pooled together and subjected to HTS.

The HTS generated 132,145,560 raw reads totaling 19.822 GB bases. After filtering out low-quality reads and adapters, 124,008,416 clean reads remained. Reads that mapped to the loquat genome were excluded, leaving 22.9% (28,399,384 reads) unmapped, which were de novo assembled into contigs using Trinity software 9 (Cambridge, MA, USA). A subsequent BLASTn analysis using these contigs as queries revealed significant matches to viral sequences; 28 contigs ranging from 520 to 2939 nt mapped to tomato mosaic virus (ToMV) reference sequence provided 93.2% genome coverage. There were 32 contigs ranging from 77 to 248 nt mapped to the citrus exocortis viroid (CEVd) reference sequence, covering 98.5% of its genome. These findings indicate the presence of both ToMV and CEVd in the loquat samples.

To confirm the infection of ToMV and CEVd, total RNA was extracted from the three samples used for HTS, and reverse transcription polymerase chain reaction (RT-PCR) was performed using specific primer pairs (Table S1) designed based on the assembled contigs. PCR products with expected sizes (480 and 361 bp) were obtained in all three samples, indicating that all three loquat plants were co-infected with ToMV and CEVd.

### 2.2. Whole Genome Sequence and Characterization of ToMV-Loquat Isolate

To obtain the full-length genomic sequence of ToMV from the loquat, specific primers (referenced in Table S1) were designed; RT-PCR was performed to amplify fragments of ToMV genomic RNA, and the 5′ and 3′-terminal sequences were determined. The sequences of the obtained fragments covering the whole genome were assembled with SnapGene software, resulting in the complete ToMV genomic RNA. This isolate was named ToMV-Pi20 (GenBank No. PP915779).

**Figure 2:**
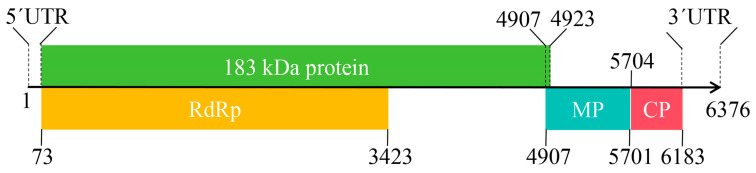
Diagrammatic representation of the ToMV-Pi20 isolate genome with nucleotide coordinates. UTR, untranslated region; RdRp, RNA-dependent RNA polymerase; MP, movement protein; CP, coat protein.

The complete genome sequence of the ToMV-Pi20 is composed of a positive-sense single-stranded RNA segment with 6376 nt in length (Figure 2). The 5′ UTR and 3′ UTR consisted of 72 and 193 nucleotides, respectively. It consists of distinct regions and four ORFs. ORF1 (nt 73–4923) is predicted to be synthesized through the read-through product of the RdRp, which consists of 4851 nucleotides and encodes a 183 kDa protein of 1617 aa residues. Both RdRp and 183 kDa proteins are involved in viral replication. ORF2 spanning nucleotide positions 73 to 3423, which covers 3351 nucleotides and encodes a 126 kDa RdRp protein comprising 1117 amino acids (aa). ORF3 started at position 4907 nt and ended at position 5701 nt, with a TAA stop codon encoding MP of 264 aa and an estimated molecular weight of 30 kDa. ORF4, spanning position 5704 to 6183 nt, encoded a putative CP of 159 aa of 17.5 kDa.

### 2.3. Homology and Variation between ToMV-Loquat and Other Isolates

ToMV-loquat was compared with 11 ToMV isolates obtained in GenBank (GW2, mutoko, SL-1, 99-1, Penghu, Queensland, NPPO-NL 41833930, INIFAP JM1, NVWA5785660, DTT, IFA9), the results indicated that the aa sequence of the 183 kDa protein in ToMV-loquat exhibited 99.4% to 99.7% identity with the corresponding genes of the other ToMV isolates. The RdRp showed a 99.7–99.9% aa identity, and the MP showed 98.9–99.6%. The CP of the ToMV-loquat had an aa sequence identity between 98.7 and 100% (Table 1).

**Figure 3:**
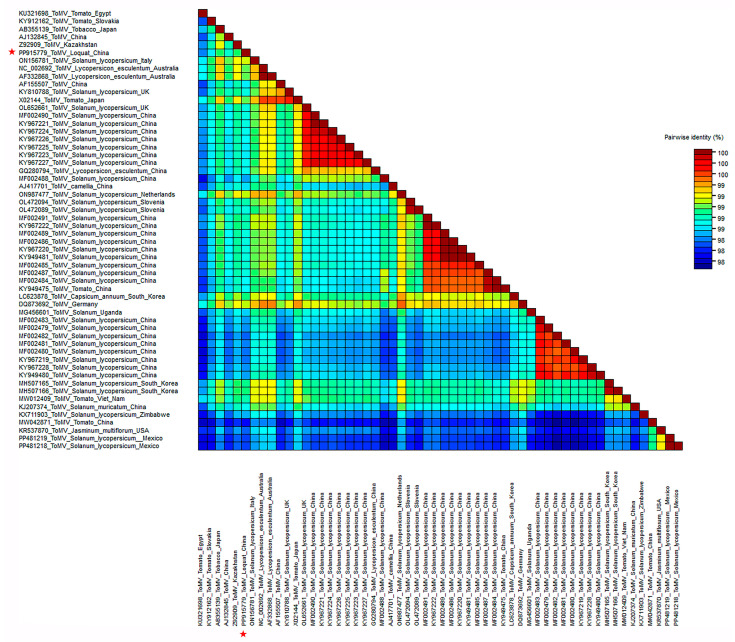
The distribution of pairwise identity scores of ToMV complete sequences aligned by MAFFT and displayed using SDT software (v1.2). The red star indicated the ToMV isolated from loquat in this study.

To further determine the relationship between ToMV-loquat and other ToMV isolates, the pairwise alignments of ToMV complete sequences were conducted using the MAFFT program. The SDT software (v1.2) was used to display the pairwise identity scores. The comparison results showed that the complete genomic sequence of the ToMV-loquat had a nucleotide identity of between 98.5 and 99.3% with other ToMV isolates and had the highest homology with the strain of ToMV-IFA9 isolated from *Solanum lycopersicum* (Tomato) in Italy and ToMV-NPPO-NL 41833930 from *Solanum lycopersicum* (Tomato) in the Netherlands (Figure 3 and Table S2).

### 2.4. Phylogenetic Relationship between ToMV-Loquat and Other ToMV Isolates

**Figure 4:**
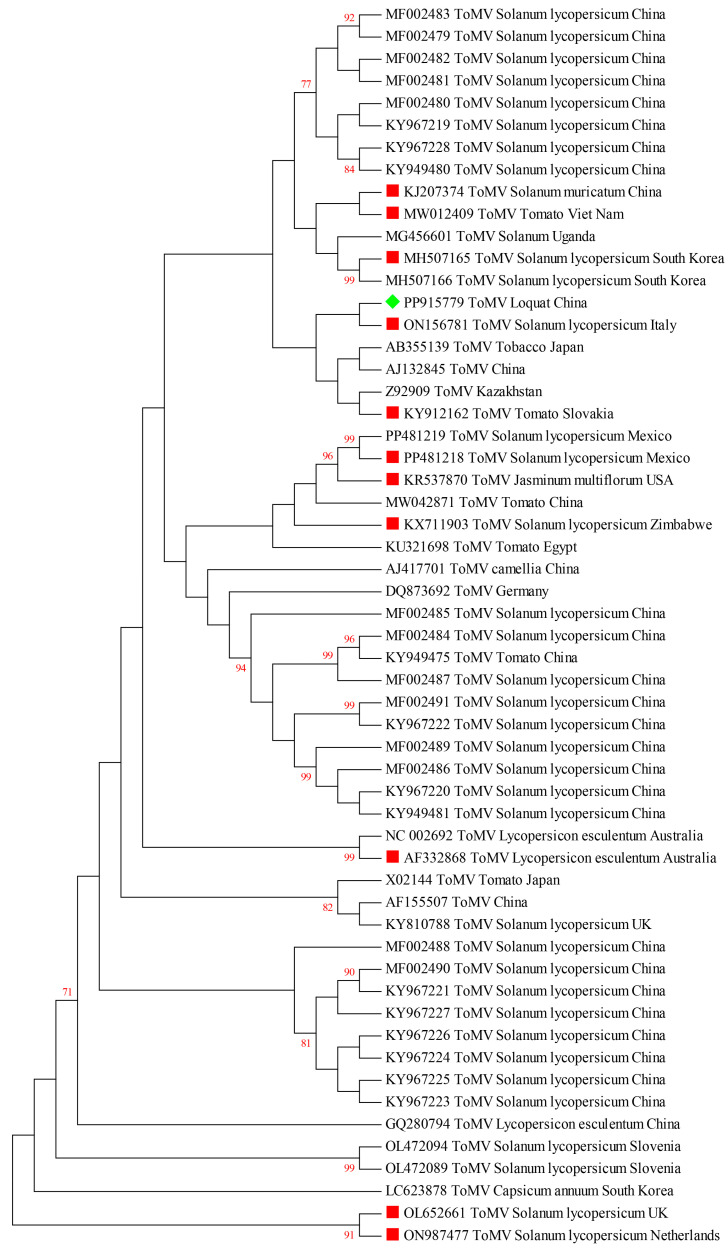
Phylogenetic relationship of ToMV-loquat and other ToMV isolates from different countries and hosts based on the complete genome sequence. The phylogenetic tree was constructed using the maximum likelihood method in MEGA 7.0, with a bootstrap of 1000 replicates (bootstrap values > 70% are shown at the nodes). The green rhombus indicated the ToMV isolated from loquat in this study, and the red squares indicated the ToMV isolates mentioned in Table 1. The red font numbers indicate the bootstrap values.

To analyze the phylogenetic relationship between ToMV-loquat and other isolates, multiple alignments of the complete sequences of different ToMV isolates were performed using MEGA 7.0. The evolutionary tree demonstrated that ToMV-loquat shares its closest evolutionary proximity with isolates IFA9 (GenBank No. ON156781), K2 (GenBank No. Z92909), SL-1 (GenBank No. KY912162), L11Y (GenBank No. AB355139), and S-1 (GenBank No. AJ132845). These isolates form a distinct cluster indicating a close genetic relationship with ToMV-loquat. In contrast, ToMV-loquat shows a more distant relationship with I NPPO-NL 41833930 (GenBank No. ON987477) from *Solanum lycopersicum* in the Netherlands, as well as isolate NVWA5785660 (GenBank No. OL652661) from *Solanum lycopersicum* in the Netherlands and the United Kingdom, which are positioned on separate branches of the phylogenetic tree (Figure 4).

### 2.5. Characterization of CEVd in Loquat

Using the specific primers CEVd-F/R, which were designed based on the HTS contigs, a CEVd sequence with 361 nt in length was amplified and named CEVd-pa24 (GenBank No. PP915780). The nucleotide sequence alignment of CEVd-loquat with other CEVd isolates indicates that CEVd-loquat shared the highest identity of 80.1% with CEVd-balad (GenBank No. PP869624) and, subsequently, CEVd-CNU_JC (GenBank No. LC758578), with 79.8%, CEVd-OH19-3 (GenBank No. MT815905), with 78.8%, CEVd-2/3 (GenBank No. DQ318790), CEVd (GenBank No. FJ662762), and CEVd-LSS OH19 (GenBank No. MT561434), with 78.3%, CEVd-5/5 (GenBank No. DQ318793), with 78.1%, CEVd-Aan-Saladin (GenBank No. OR589765), with 77.8%, CEVd-Najaf (GenBank No. OR589765), with 77.8%, and CEVd-Baghdad-1/Iraq (GenBank No. OR343512), with 75.6% (Table 2).

**Figure 5:**
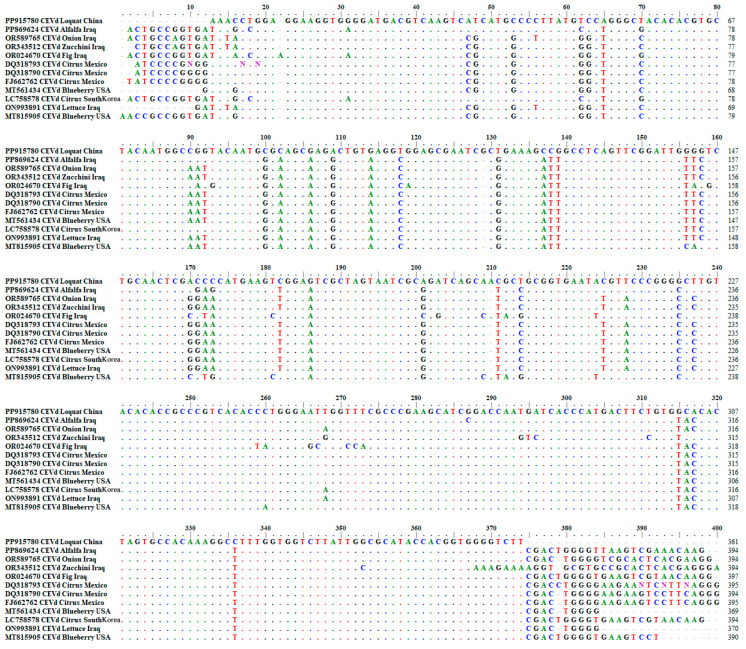
Comparison results of CEVd-loquat with reported CEVd sequences.

A multiple-sequence alignment based on CEVd-loquat and other CEVd isolates from alfalfa (PP869624), onion (OR589765), zucchini courgette (OR343512), fig (OR024670), lemon (DQ318793), sweet orange (DQ318790 and FJ662762), blueberry (MT561434 and MT815905), mandarin orange (LC758578), and lettuce (ON993891) showed that CEVd-loquat has 34–56 nucleotide substitutions and 10–35 deletion sites compared with other isolates, and the variation sites are concentrated mainly at the ends of the sequence (Figure 5).

**Figure 6:**
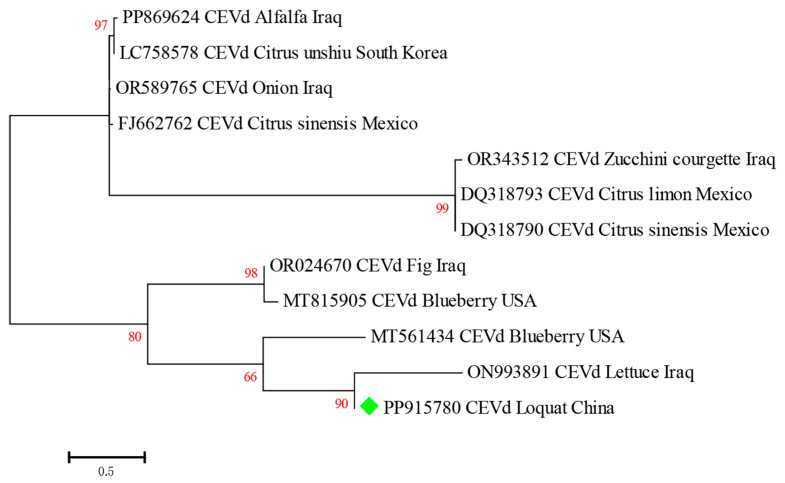
Phylogenetic relationship of CEVd-loquat and other isolates from different countries and hosts based on the nucleotide sequence. The phylogenetic tree was constructed using the maximum likelihood method in MEGA 7.0, with a bootstrap of 1000 replicates. The green rhombus indicated the CEVd isolated from loquat in this study. The red font numbers indicate the bootstrap values.

In addition, multiple alignments of nucleotide sequences of different CEVd isolates were performed using ClustalW, and then the phylogenetic trees were constructed using the maximum likelihood method with a bootstrap of 1000 replicates in MEGA 7.0. A phylogenetic analysis indicated that all CEVd isolates could be grouped into four main clades. The Clade I virus isolates were PP869624 CEVd Alfalfa Iraq, LC758578 CEVd Citrus unshiu South Korea, OR589765 CEVd Onion Iraq, and FJ662762 CEVd Citrus sinensis Mexico. Three isolates (OR343512 CEVd Zucchini courgette Iraq, DQ318793 CEVd Citrus limon Mexico, and DQ318790 CEVd Citrus sinensis Mexico) were grouped into the Clade II and were closely related. OR024670 CEVd Fig Iraq and MT815905 CEVd Blueberry USA were grouped into Clade III. CEVd-loquat isolated in this study was closely related to ON993891 CEVd-Lettuce Iraq and MT561434 CEVd Blueberry USA and fell into Clade IV (Figure 6).

## 3. Discussion

Plant virus diagnostics using traditional approaches has its drawbacks, such as long detection times, high technological requirements, and difficulty in detecting viruses with significant sequence variations or unknown viruses. With the ability to identify and characterize both known and unknown viruses infecting a plant, HTS has developed into a potent tool in plant pathology research, providing a comprehensive view of the plant virome [19]. This study identified a virus ToMV and a viroid CEVd in loquat leaves exhibiting symptoms of viral disease using HTS and confirmed by RT-PCR. As far as we know, this is the first instance of ToMV and CEVd infection in loquat, providing new information about the host range of this virus and viroid.

ToMV is a positive-sense single-stranded RNA virus, primarily transmitted through plant-to-plant contact via sap, while infected seeds and fruits serve as the main vectors for long-distance transmission. Since it was first reported in the United States in 1909, ToMV has been reported on more than five species, including tomato, tobacco, *Jasminum multiflorum*, camellia, and *Capsicum annuum*; it has become one of the globally widespread viruses. This study obtained the complete genome sequence of ToMV isolated from loquat, and the analysis revealed that this isolate shares nucleotide sequence similarity with other isolates of over 98.5% and had the highest homology with the strains of ToMV-IFA9 isolated from *Solanum lycopersicum* (tomato), reaching 99.3%. A systematic evolutionary analysis indicates that ToMV-loquat is most closely related to the Italian isolates IFA9, suggesting that this virus lacks geographical specificity and provides insights into the genetic diversity and evolutionary relationships within the ToMV species, highlighting specific strains closely related to ToMV-loquat and those that are more genetically distant. Unlike most viruses, which are primarily transmitted through sap for aphids, ToMV can also spread through fruits and seeds [20]. This presents a challenge for the virus’s control and prevention. In production, it is necessary to employ control strategies that include both the prevention of insect pests and the treatment of diseases while also avoiding virus spread caused by the sale of infected fruits.

This study, through HTS analysis conducted on diseased loquat samples, for the first time identified loquat as a new natural host of CEVd, which is primarily known to infect citrus. The circular genome of the CEVd-loquat isolate was amplified and characterized. CEVd-loquat showed identities less than 80.1% with other isolates, indicating a high sequence variability in CEVd, which may be associated with its host adaptation. A multiple sequence alignment comparing CEVd-loquat with other CEVd isolates from various hosts revealed the presence of 34–56 nucleotide substitutions and 10–35 deletion sites between CEVd-loquat and these isolates. These mutation sites illustrate the genetic diversity and unique evolutionary path of CEVd-loquat compared to its counterparts isolated from different plant hosts. Understanding these genetic variations is crucial for studying the adaptation, transmission, and management strategies of CEVd in diverse agricultural settings.

Due to the absence of infectious clones for ToMV and CEVd and the co-infection of loquat samples collected in this study with ToMV and CEVd, the pathogenicity of these viruses and viroids cannot be validated on loquats or model plants using infectious clones or mechanical inoculation. The current results are insufficient to determine whether the symptoms observed in field plants are caused by either of them or both. Further research is needed to expand the sample collection range to clarify their incidence and pathogenicity on loquats. This study enriches the information on isolates of ToMV and CEVd, providing a basis for investigating the population genetic characteristics, sequence variation, evolution, geographical distribution, and genetic diversity of ToMV and CEVd.

The management of ToMV and CEVd often involves using certified virus-free seeds, practicing strict sanitation measures to prevent mechanical transmission, and employing resistant cultivars when available. Additionally, controlling water sources to prevent virus spread through irrigation is crucial in managing the disease, particularly in agricultural and greenhouse settings.

Remarkably, our analysis sheds new light on the spectrum of viral and viroid infections impacting loquat. This newfound knowledge not only enhances our understanding of disease dynamics within loquat cultivation but also paves the way for informed strategies aimed at mitigating the impact of viral and viroid infections on loquat production.

## 4. Materials and Methods

### 4.1. Sample Collection and RNA Isolation

Loquat leaves showing virus-like symptoms (yellowing, blistering, mosaic, leaf upward cupping, crinkle, and leaf narrowing) were collected from 2-year-old plants in a private orchard from the city of Panzhihua in Sichuan Province, China, in November 2023. RNA was extracted from loquat leaves using different methods, depending on its intended use. Total RNAs used for RT-PCR detection were extracted from 0.1 g loquat leaves by TRIzol reagent (Invitrogen, Shanghai, China) following the manufacturer’s instructions. For HTS, total RNAs were extracted using RNAprep Pure Plant Plus Kit (Tiangen, Beijing, China).

### 4.2. cDNA Library Construction and RNA-Seq

The ribosomal RNA was depleted from the total RNA obtained in Section 4.1 using an Epicentre Ribo-Zero™ rRNA Removal Kit (Epicentre, Madison, WI, USA) resulting in the ribo-depleted RNA sample. A cDNA library was constructed using NEBNext^®^ Ultra™ Directional RNA Library Prep Kit for Illumina^®^ (NEB, Beijing, China). Then, the library was subjected to high-throughput sequencing using the Illumina Hiseq 4000 with a paired-end 150-bp setup (Novogene, Tianjing, China).

### 4.3. Sequence Assembly of ToMV and CEVd

Raw reads trimmed the adapters and filtered the host genome sequences to obtain clean reads. Clean reads were de novo assembled using Trinity (v2.2.0) as described previously [21,22]. The de novo assembly of contigs was used as queries for BLASTn and BLASTx against the NCBI databases (https://blast.ncbi.nlm.nih.gov/Blast.cgi (accessed on 20 December 2023)). The obtained contigs were analyzed and assembled with DNAMAN software, version 5.0 (Lynnon Biosoft, Quebec, QC, Canada), and SnapGene (4.1.8) software.

### 4.4. Whole Genome Sequence Amplification of ToMV

To determine the complete genome sequence, gaps were bridged, and low-quality sequences were confirmed with reverse transcription polymerase chain reaction (RT-PCR) (see Section 4.6) using virus-specific primers (Table S1) designed based on the sequences of contigs; the 5′- and 3′-terminal sequences were determined using a rapid amplification of cDNA ends (RACE) 5′/3′ kit (TaKaRa, Dalian, China). Amplicons with the expected sizes were cloned into the pTOPO Blunt Simple Cloning Vector (Aidelab, Beijing, China), and at least three clones per amplicon were sent for Sanger sequencing. All viral sequences were analyzed and assembled using the SnapGene program.

### 4.5. Bioinformatics Analyses

Using ORF Finder in-line software (https://www.ncbi.nlm.nih.gov/orffinder/ (accessed on 9 February 2024)) and SnapGene (4.1.8) software to predict the ToMV-loquat genome structure, the ClustalW method was applied to multiple sequence alignments, and the phylogenetic tree was constructed using the maximum likelihood method in MEGA 7.0 [23], with a bootstrap of 1000 replicates. The sequence pair identities were calculated and aligned using the ClustalW program, and then the SDT software (v1.2) [24] displayed the pairwise identity scores using a color-coded matrix. Multiple sequence alignments of CEVd were performed using BioEdit software (v7.5.2).

### 4.6. RT-PCR Amplification, Cloning, and Sequencing

First-strand cDNAs were synthesized at 42 °C for 1 h using M-MLV reverse transcriptase (Promega, Beijing, China). The viral sequences were amplified by high fidelity Pfu DNA polymerase (Tiangen, Beijing, China) using the specific primers (Table S1) with the following reaction conditions: initial denaturation at 94 °C for 3 min, followed by 35 amplification cycles of denaturation 94 °C for 30 s, annealing at 55 °C for 30 s, and extension at 72 °C for 30 s and a final extension at 72 °C for 10 min. PCR products were subjected to 1.5% agarose gel electrophoresis, purified using the AxyPrep™ DNA Gel Extraction Kits (Axygen, Silicon Valley, CA, USA), and ligated into the pTOPO vector (Aidelab, Beijing, China). The recombinant plasmids were transformed into *Escherichia coli* DH5α cells, and positive clones were randomly selected for sequencing.

## Figures and Tables

**Table 1:** Amino acid sequence similarity comparison of the encoded proteins between ToMV-loquat and other 11 ToMV isolates.

Isolate	GenBank Accession No.	Host		Length (nt)	Identities (aa) %			
			Origin of Country		183 kDa Protein	RdRp	MP	CP
GW2	MH507166	*Solanum lycopersicum* (Tomato)	South Korea	6383	99.5	99.7	99.6	100
mutoko	KX711903	*Solanum lycopersicum* (Tomato)	Zimbabwe	6383	99.6	99.9	99.3	99.4
SL-1	KY912162	*Solanum lycopersicum* (Tomato)	Slovakia	6383	99.5	99.8	98.9	98.7
99-1	KR537870	*Jasminum multiflorum*	USA	6383	99.6	99.8	98.9	100
Penghu	KJ207374	*Solanum lycopersicum* (Tomato)	China	6385	99.4	99.8	98.9	98.7
Queensland	AF332868	*Solanum lycopersicum* (Tomato)	Australia	6383	99.7	99.9	98.9	100
NPPO-NL 41833930	ON987477	*Solanum lycopersicum* (Tomato)	The Netherlands	6373	99.6	99.9	99.3	98.7
INIFAP JM1	PP481218	*Solanum lycopersicum* (Tomato)	Mexico	6447	99.6	99.8	98.9	100
NVWA5785660	OL652661	*Solanum lycopersicum* (Tomato)	UK	6367	99.6	99.9	99.3	99.4
DTT	MW012409	*Solanum lycopersicum* (Tomato)	Vietnam	6380	99.6	99.9	99.3	100
IFA9	ON156781	*Solanum lycopersicum* (Tomato)	Italy	6376	99.6	99.9	99.6	100

**Table 2:** Nucleotide sequence comparison between CEVd-loquat and other isolates.

Isolate	GenBank Accession No.	Origin of Country	Host	Length (nt)	Identities (nt) %
Aan-Saladin	OR589765	Iraq	*Allium cepa* (Onion)	394	77.8
Baghdad-1/Iraq	OR343512	Iraq	*Cucurbita pepo* (Zucchini courgette)	394	75.6
Najaf	OR024670	Iraq	*Ficus carica* (Fig)	397	77.3
5/5	DQ318793	Mexico	*Citrus limon* (Lemon)	395	78.1
2/3	DQ318790	Mexico	*Citrus sinensis* (Sweet orange)	394	78.3
CEVd	FJ662762	Mexico	*Citrus sinensis* (Sweet orange)	395	78.3
LSS OH19	MT561434	USA	*Vaccinium uliginosum* (Blueberry)	369	78.3
CNU_JC	LC758578	South Korea	*Citrus unshiu* (Mandarin orange)	394	79.8
Tikrit	ON993891	Iraq	*Lactuca sativa* (Lettuce)	370	77.8
OH19-3	MT815905	USA	*Vaccinium uliginosum* (Blueberry)	390	78.8
Balad	PP869624	Iraq	*Medicago sativa* (Alfalfa)	394	80.1

## Data Availability

The data presented in this study and the raw data for HTS are available on request from the corresponding author.

## Supplementary Materials

The following supporting information can be downloaded at: https://www.mdpi.com/article/10.3390/plants13141965/s1, Table S1. Primers used in this study. Table S2. Nucleotide sequence comparison of ToMV complete sequences.
